# Driving delivery and uptake of catch-up vaccination among adolescent and adult migrants in UK general practice: a mixed methods pilot study

**DOI:** 10.1186/s12916-024-03378-z

**Published:** 2024-05-03

**Authors:** Alison F. Crawshaw, Lucy P. Goldsmith, Anna Deal, Jessica Carter, Felicity Knights, Farah Seedat, Karen Lau, Sally E. Hayward, Joanna Yong, Desiree Fyle, Nathaniel Aspray, Michiyo Iwami, Yusuf Ciftci, Fatima Wurie, Azeem Majeed, Alice S. Forster, Sally Hargreaves

**Affiliations:** 1 Migrant Health Research Group, Institute for Infection and Immunity, St George’s, University of London, London, UK; 2 Population Health Research Institute, St George’s, University of London, London, UK; 3 Faculty of Public Health and Policy, London School of Hygiene and Tropical Medicine, London, UK; 4 NHS North Central London Research Network (NoCLoR) and Clinical Research Network (CRN) North Thames, London, UK; 5 Addiction and Inclusion Directorate, Office for Health Improvement and Disparities, Department of Health and Social Care, 39 Victoria Street, London, SW1H 0EU UK; 6 Department of Primary Care and Public Health, Imperial College London, London, UK; 7 Our Future Health, Manchester, UK

**Keywords:** Vaccination, Catch-up vaccination, Primary care, Migrants, Transients and migrants, Health inequalities, Vaccine-preventable diseases, Immunisation, Health systems

## Abstract

**Background:**

Migrants in the UK and Europe face vulnerability to vaccine-preventable diseases (VPDs) due to missed childhood vaccines and doses and marginalisation from health systems. Ensuring migrants receive catch-up vaccinations, including MMR, Td/IPV, MenACWY, and HPV, is essential to align them with UK and European vaccination schedules and ultimately reduce morbidity and mortality. However, recent evidence highlights poor awareness and implementation of catch-up vaccination guidelines by UK primary care staff, requiring novel approaches to strengthen the primary care pathway.

**Methods:**

The ‘Vacc on Track’ study (May 2021–September 2022) aimed to measure under-vaccination rates among migrants in UK primary care and establish new referral pathways for catch-up vaccination. Participants included migrants aged 16 or older, born outside of Western Europe, North America, Australia, or New Zealand, in two London boroughs. Quantitative data on vaccination history, referral, uptake, and sociodemographic factors were collected, with practice nurses prompted to deliver catch-up vaccinations following UK guidelines. Focus group discussions and in-depth interviews with staff and migrants explored views on delivering catch-up vaccination, including barriers, facilitators, and opportunities. Data were analysed using STATA12 and NVivo 12.

**Results:**

Results from 57 migrants presenting to study sites from 18 countries (mean age 41 [SD 7.2] years; 62% female; mean 11.3 [SD 9.1] years in UK) over a minimum of 6 months of follow-up revealed significant catch-up vaccination needs, particularly for MMR (49 [86%] required catch-up vaccination) and Td/IPV (50 [88%]). Fifty-three (93%) participants were referred for any catch-up vaccination, but completion of courses was low (6 [12%] for Td/IPV and 33 [64%] for MMR), suggesting individual and systemic barriers. Qualitative in-depth interviews (*n* = 39) with adult migrants highlighted the lack of systems currently in place in the UK to offer catch-up vaccination to migrants on arrival and the need for health-care provider skills and knowledge of catch-up vaccination to be improved. Focus group discussions and interviews with practice staff (*n* = 32) identified limited appointment/follow-up time, staff knowledge gaps, inadequate engagement routes, and low incentivisation as challenges that will need to be addressed. However, they underscored the potential of staff champions, trust-building mechanisms, and community-based approaches to strengthen catch-up vaccination uptake among migrants.

**Conclusions:**

Given the significant catch-up vaccination needs of migrants in our sample, and the current barriers to driving uptake identified, our findings suggest it will be important to explore this public health issue further, potentially through a larger study or trial. Strengthening existing pathways, staff capacity and knowledge in primary care, alongside implementing new strategies centred on cultural competence and building trust with migrant communities will be important focus areas.

**Supplementary Information:**

The online version contains supplementary material available at 10.1186/s12916-024-03378-z.

## Background

Adult migrants arriving in the UK and Europe may be vulnerable to vaccine-preventable diseases (VPDs) as a result of childhood immunisation gaps and marginalisation from health systems, requiring alignment with national immunisation schedules [1–4]. A global scoping review reporting data from 45 studies in high- and high-middle income countries concluded that migrants generally experience higher VPD burden and lower immunisation rates compared to non-migrant populations [5]. Migrant populations, particularly those residing in camps and temporary housing, have also been recognised as an at-risk group susceptible to outbreaks of VPDs like measles in Europe [6]. Moreover, the COVID-19 pandemic has exacerbated immunisation disparities globally, by impacting on the delivery of routine immunisation programmes [7]. A recent analysis of 125,526 refugees in an IOM resettlement programme to the UK revealed concerning figures, with only 11% of refugees fully aligned with the UK schedule for polio, 34% for measles, and 5% for diphtheria and tetanus; notably, adults were more likely to be under-vaccinated than children [3]. Although the possibility of adults having acquired natural immunity to VPDs is a consideration, a recent review and meta-analysis reported immune coverage well below herd immunity thresholds for life-threatening VPDs including diphtheria and measles among migrants in the European region, indicating that there is still a need for catch-up among these groups [8]. Consequently, there is a compelling case for offering catch-up vaccinations to migrants in UK primary care upon arrival and opportunistically, particularly considering that adolescent and adult migrants are often overlooked in vaccination programmes upon entering European countries [9]. Despite clear guidelines for catch-up vaccinations, awareness and implementation remain low in practice [10]. Better understanding of the barriers and facilitators to catch-up vaccination among mobile and migrant populations is urgently needed, aiming to achieve immunisation coverage targets and ensure equitable access to vaccines [11].

The documented factors contributing to the risk of under-immunisation among some migrants for routine vaccinations are diverse and multi-faceted [12–15]. These encompass cultural, socio-structural, political, economic, and behavioural elements, often compounded by language barriers and a lack of specific procedures for engaging older age groups (as opposed to children) in catch-up vaccination. Migrants may come from countries with differing immunisation schedules, reduced availability of vaccines, poor health system infrastructure, or fragmented delivery systems. As such, these populations may have missed essential vaccines, doses, boosters, and the introduction of newer vaccines, such as human papillomavirus (HPV) and meningococcal vaccines, not available in their home countries. In both host and destination countries, reluctance to engage with vaccination services can stem from mistrust of healthcare systems, public health and immigration authorities, governmental bodies, racism, discrimination, physical access barriers, and specific vaccine-related beliefs or concerns. Unfortunately, the burden often falls on migrants to navigate healthcare systems, emphasising the need for more equitable and inclusive approaches and policies that actively involve these groups. Unlike migrant children, who typically catch up on missed childhood vaccines through the school system, adult and adolescent migrants face heightened vulnerability to remaining unvaccinated during and after migration due to weak systems for checking vaccination history, offering catch-up vaccinations, and inconsistent implementation and interpretation of guidance.

**Fig. 1:**
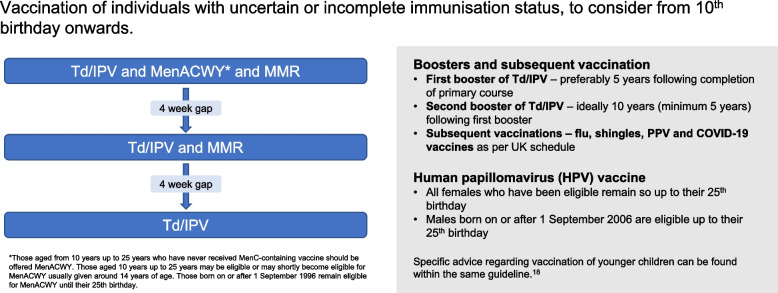
Vaccination of individuals with uncertain or incomplete immunisation status, to consider from 10th birthday onwards. Reproduced from [18]. Guidance states unless there is a documented or reliable verbal vaccine history, individuals should be assumed to be unimmunised and a full course of immunisations planned. MMR=measles, mumps, rubella; Td/IPV= tetanus, diphtheria, polio; HPV= human papillomavirus vaccine; PPV=pneumococcal vaccine; MenACWY= meningococcal conjugate vaccine. Guidance for other age groups is provided in the original source

Various frameworks have been established to improve vaccination coverage for VPDs and to ensure equitable access and uptake of vaccinations among migrant populations [11, 16–19]. The World Health Organization’s (WHO) Immunization Agenda 2030 (IA2030) outlines strategic priorities aimed at strengthening immunisation in primary care, expanding equitable access to vaccination for vulnerable populations, and incorporating catch-up vaccination for missed vaccines and doses across the life-course. IA2030 advocates for the implementation of catch-up vaccination policies and schedules in all countries, offering specific guidance [11], as does the European Centre for Disease Prevention and Control (ECDC) [20] for European nations. In the UK, the UK Health Security Agency (UKHSA) provides explicit guidance, often referred to as an algorithm, for vaccinating individuals with uncertain or incomplete immunisation status [21] (Fig. 1).﻿ According to the guidance, catch-up vaccinations should be part of routine care and include measles, mumps, rubella (MMR), tetanus, diphtheria, polio (Td/IPV), HPV (aged 11–25 years), and meningococcal (MenACWY) (aged 10–25 years) vaccines [21]. This guidance is particularly relevant to many migrants who often lack vaccine records or recorded childhood vaccine history in primary care systems. However, the effective implementation of this guidance in primary care is deficient [10], with adult migrants frequently excluded from catch-up vaccination initiatives due to the absence of established pathways for engaging these individuals upon their presentation to primary care and a lack of knowledge among frontline staff regarding effective approaches [10]. Additionally, data on migrant status, including overseas-born status and country of origin, are not routinely coded into electronic patient records in UK primary care, limiting our ability to detect gaps in vaccination coverage and address catch-up vaccination needs for specific migrant groups.

We therefore did a prospective, observational mixed-methods pilot study, ‘Vacc on Track’, to measure rates of under-vaccination among adolescent and adult migrants presenting to UK primary care and to better understand and define new referral pathways for catch-up vaccination, ahead of a potential larger-scale trial.

## Methods

### Study design and procedure

We conducted a prospective, observational mixed-methods pilot study from May 2021-September 2022 in seven GP practices across two urban London boroughs. The study was designed as a pilot to test processes and approaches which may inform a future large-scale study or trial. The overall objectives were to measure routine vaccination coverage among migrants presenting to UK primary care and establish and test new referral pathways for catch-up vaccination. The study procedure was as follows: following recruitment, participants were asked about their vaccination history (including for routine childhood immunisations including MMR, Td/IPV, and other vaccines including tuberculosis/bacille Calmette-Guerin vaccine (TB/BCG) and HPV), which was coded into their electronic patient record and the study database. In the absence of a written vaccination card or record documenting a completed vaccine course, or if patients said they had not had a vaccine or were unsure, patients were referred for catch-up vaccination for each eligible vaccine (following the UK algorithm for vaccinating individuals with uncertain or incomplete immunisation status [21]) and invited to attend an appointment(s) with their practice nurse. Eligible catch-up vaccines were MMR, Td/IPV, HPV (aged 11–25 years) and MenACWY (aged 10–25 years). Practice nurses followed the UK algorithm to administer missing vaccine doses, boosters, and courses and recorded the data into the patient’s electronic record and the study database. A standardised data collection tool was designed to facilitate the collection of data, which then prompted referrals for catch-up vaccination (see *Data collection and referral pathway for catch-up vaccination*).

PICOTS criteria for the study are shown in Table 1. In addition to collecting quantitative data from migrant patients, we explored the views of practice staff on catch-up vaccination and current guidance, including barriers to implementation, suggestions, and areas for improvement and support, through focus group discussions (FGDs), which were carried out in August 2022. During the study, we also decided to conduct an in-depth interview with two staff members to explore examples of good practice from the most successful (in terms of recruitment and uptake) participating practice. We carried out in-depth interviews with a diverse range of recently arrived (≤ 10 years) migrants to explore views and concerns around catch-up vaccination after arrival in the UK. The study tool, recruitment, and data collection pathways are shown in Fig. 1. The reporting of this study follows the Strengthening the Reporting of Observational Studies in Epidemiology (STROBE) guidelines [22].

**Table 1:** PICOTS criteria

**P**atients	Adult and adolescent migrant patients (≥ 16 years), born outside of Western Europe, North America, Australia, and New Zealand
**I**ntervention	Novel referral pathways and new standardised data collection tool
**C**ontrol	None
**O**utcomes	Uptake of MMR, Td/IPV, MenACWY, HPV, and other routine vaccinations; Self-reported previous history of VPDs; Rates of under-vaccination for routine VPDs; Acceptability, views, and practices from practice staff Views and experiences of recently arrived migrants on catch-up vaccination in primary care
**T**ime	Up to 14 months (minimum 6 months)
**S**tudy design	Prospective observational study in 2 boroughs (7 GP practices) in London, UK

### Ethics and PPI

This study received ethics approval from the NHS Health Research Authority Yorkshire and Humber—South Yorkshire Research Ethics Committee (20/YH/0342) on 18 December 2020. The qualitative in-depth interview study with migrants received ethics approval from the St George’s, University of London Research Ethics Committee (REC reference: 2020.0058). Migrants with lived experience of the UK immigration and healthcare systems were involved in the design of this study through our National Institute for Health and Care Research (NIHR)-funded Patient and Public Involvement and Engagement (PPIE) Project Advisory Board and were compensated for their time and contributions.

### Population, recruitment, and informed consent process

The study was conducted with support from the NHS North Central London Research Network (NoCLoR) and the North Thames Clinical Research Network (CRN). GP practices in areas with a high proportion of migrant residents were purposively invited to join the study. We aimed to recruit up to 10 GP practices across two boroughs (Barnet and Tower Hamlets) in North and East London (referred to henceforth as sites 1 and 2), with a target sample size of 100 participants. Boroughs were selected for their high proportion of migrant residents (estimated to be approximately half, according to 2021 Census data [23]). Both rank in the top 50% of most deprived local authorities in England, based on the English indices of deprivation 2019 [24], although Tower Hamlets ranks as significantly more deprived than Barnet. In practice, seven GP practices were recruited, with six across site 1 and one practice belonging to site 2.

**Fig. 2:**
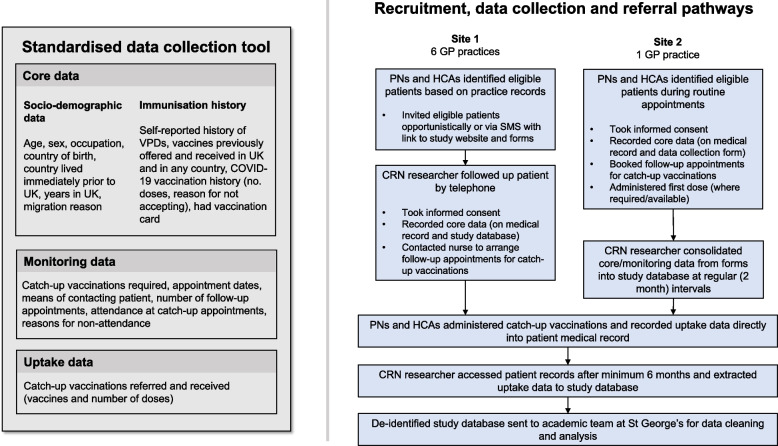
Figure showing standardised data collection tool (left) and referral pathways implemented in study sites 1 and 2 (right). VPDs, vaccine-preventable diseases; PN, practice nurse; HCA, healthcare assistant; CRN, clinical research network

Patients registered at participating practices were eligible for the study if they were (a) aged 16 years or older, (b) born outside of the UK (our migrant definition excluded those born in North America, Australia, New Zealand, or Western Europe, as defined by the UN maximal definition of Western Europe [25]), and (c) capable of giving informed consent. Recruitment procedures differed between the two sites (see Fig. 2).

We held site initiation visits with all practice sites, inviting GPs, practice managers, healthcare assistants (HCAs), and nurses involved in immunisation. Alongside delivering training on the current UK primary care catch-up vaccination guidelines [21] and the referral pathway to implement upon identifying under-vaccinated patients, these visits covered the study processes and procedures, approaches to identifying the study population and recruiting participants, and use of the standardised data collection tool.

At site 1 (*n* = 6 GP practices), clinical practice staff were originally going to recruit and consent patients. However, the recruitment pathway was modified as clinical staff were under intense pressure from the COVID-19 pandemic, so the CRN led the recruitment and consenting process. Practice nurses and HCAs first identified patients who met the eligibility criteria, filtering patient records by ethnicity or notes on migrant status (where recorded) to identify those potentially eligible and sent an SMS/text message with a link to the study website, from which patients could download the study documents (participant information sheet [PIS], consent form, and leaflets about catch-up vaccination and HPV vaccination, all available in the six dominant local languages, which were Arabic, Farsi, Pashtu, Romanian, Urdu, English). A researcher at the CRN (DF) then followed up with patients by a telephone call enquiring whether they would like to join the study and to take informed consent. Practice nurses also mentioned the study opportunistically to patients during routine appointments, who would then be referred to the CRN researcher (DF) for consent. At site 2 (*n* = 1 GP practice), the practice nurses HCAs invited and consented participants to the study opportunistically during routine appointments, as per the original recruitment pathway. Participants were given hard copies of the study documents and given the opportunity to ask questions and decide whether they wanted to participate. We gave practice and CRN staff a copy of a form detailing the names of common childhood vaccines in multiple languages, to support taking vaccine history during appointments (see Supplementary files). Telephone interpreters (via Language Line) were available on request at both sites during recruitment and data collection.

### Data collection and referral pathway for catch-up vaccination

We developed a standardised data collection tool using Microsoft Excel, which was used to collect specific sociodemographic information (such as country of birth, which is not routinely recorded in patient records), immunisation history, and monitoring and uptake data when patients were referred for catch-up vaccination (Fig. 2). We documented participants’ rates of under-vaccination for MMR, Td/IPV, and other key vaccines in the UK routine immunisation schedule, history of VPDs, and uptake rates of MMR, Td/IPV, MenACWY, and HPV vaccines following referral to the practice nurse for catch-up vaccination. We also explored sociodemographic factors associated with under-vaccination in the study population. Immunisation history was based on self-reporting or vaccination records (via the primary care computer system or hand-held vaccination cards) where available.

Data collection and referral pathways and procedures differed between sites and are outlined in Fig. 2. In site 1, the CRN researcher collected core data via telephone call with the participant, which were recorded in the patient’s electronic medical record and on the password-protected study database. The CRN researcher determined the participant’s need for catch-up vaccinations based on the study training and the UK catch-up vaccination guidelines [21] and, if accepted by the participant, contacted the practice nurse (at the practice where the patient was registered) to arrange an appointment. Once the CRN staff had facilitated an appointment for first doses, they then left practice nurses to follow-up patients for subsequent doses as per routine care. Subsequent catch-up vaccination doses (uptake data) were recorded by practice staff in the patient’s medical record at the time of administration and these data were later extracted by the CRN researcher (see *Data management, follow-up, and statistical analysis*). In site 2, the practice nurse collected core data (recorded in the patient’s medical record) during face-to-face appointments, administered first doses where vaccine stocks allowed, and booked patients for any necessary follow-up appointments for catch-up vaccinations and subsequent doses. Anonymised study data (core, monitoring and uptake data) were extracted from electronic patient records by the practice manager at site 2 and securely transferred to the CRN researcher, who added them to the aggregate study database.

### Data management, follow-up, and statistical analysis

We aimed to follow-up patients for a minimum of 6 months at both sites to allow for all doses (Td/IPV is 3 doses, with a 4-week gap in between each; Fig. 1). At the end of follow-up, the CRN researcher securely extracted monitoring and outcomes data from participants’ electronic medical records and updated the aggregate study database. A de-identified, anonymised version was then transferred securely to the study team at St George’s for data cleaning and analysis.

Data cleaning and analyses were done using STATA 12. All tests were two-tailed and *p* values less than 0.05 were regarded as significant. We used descriptive statistics to describe the sociodemographic characteristics, vaccination history, VPD history, and catch-up vaccine uptake of participants. We summarised continuous data with mean and standard deviation (SD) and described categorical responses using the frequency and percentage. Comparisons between categorical variables were calculated using Pearson’s chi-squared test, and comparisons between continuous variables were calculated using unpaired *t*-tests.

Bivariable and multivariable logistic regression analyses were chosen to model the relationship between a binary outcome and predictor variables and were used to look for factors associated with being un-vaccinated (received zero doses) or under-vaccinated (received at least 1 dose, but not full schedule) for key vaccines at the time of study enrolment. Outcomes included un-vaccinated for MMR vaccine, un-vaccinated for Td/IPV vaccine, un-vaccinated for MMR vaccine *and* Td/IPV vaccine, unvaccinated for any polio—combined or single vaccines, unvaccinated for any measles—combined or single vaccines, and under-vaccinated for MMR vaccine or Td/IPV vaccine. Explanatory variables were age, sex, birth region, region lived prior to the UK, years in the UK, and study site (migration reason and occupation were only recorded in site 1 and were therefore not included in the regression analyses). Multivariable models were built in a forward, stepwise fashion. Age, sex, and birth region were adjusted for in each multivariable model; certain variables were removed from the final model to reduce collinearity.

### Qualitative component

Our qualitative component included FGDs and an in-depth interview conducted with practice staff from participating practices and in-depth interviews conducted with recently arrived migrants. Topic guides were developed by the research team. The interviews with migrants were done remotely (either over the phone or through video call) across 17 months. Migrant participants were recruited using purposive and snowball sampling, with the aim of recruiting participants from a broad range of nationalities, migration statuses, and age groups. Adverts for the study and participant information sheets were circulated to 20 UK-based migrant support groups (mostly based in South London and chosen for their locality around St George’s, University of London) and on social media. Those who expressed an interest in taking part were contacted by telephone, and the study was explained to them with interpreters available on request. Translated participant information sheets were circulated, and written informed consent was obtained from all participants prior to carrying out an interview (methods reported in full elsewhere [26]). We did three FGDs which were scheduled to take place at the end of routine practice meetings conducted on Microsoft Teams (most convenient for participants). Participants were practice nurses, HCAs, and practice managers (roles involved in vaccination delivery/scheduling) from the participating practices. An in-depth interview was conducted with two staff from site 2 (due to timing, these staff had not participated in FGDs). For the FGDs, all staff received information about the study and how their data would be used in advance, which was reiterated at the start of the meeting, and staff were able to make an informed decision about their participation. Participants were asked to imply consent by remaining on the call, which was considered appropriate because the topic was low risk, not audio recorded, and anonymised summary feedback (broad views) was collected. All participants received a PIS and provided written informed consent prior to participating. Both the FGD and staff interviews followed a semi-structured topic guide, which explored participants’ experiences of implementing the study, current barriers and challenges to delivering catch-up vaccinations, and suggestions for improving the tool, referral pathways, and engaging migrant patients/promoting catch-up vaccination among these groups. Broad views and selected short-hand quotations (non-attributable) were collected during FGDs in the form of hand-written and typed notes (by SH and LPG). The staff interview was conducted by AFC with two staff participants in a private room, audio-recorded and transcribed verbatim by a professional transcription service.

Qualitative data were analysed deductively using a flexible and rapid thematic analysis and evaluation approach [27]. Notes from the FGDs which were reflected on and discussed afterwards by AFC, SH and LPG, and AFC then independently coded and grouped the findings into broad barrier and facilitator concepts using a matrix method (by hand and in Microsoft Excel). The data in the matrix were corroborated and discussed again by the three researchers, to ensure rigour and coding reliability. The same approach was used to analyse the transcript of the key informant interview. Migrant interviews were analysed using the thematic framework approach in NVivo 12. Triangulation occurred when the qualitative and quantitative data were combined but also by the interaction between the three researchers during data collection and analysis and through the contributions of their own perceptions, beliefs, and academic disciplines to the collection and interpretation of data.

## Results

We recruited 57 migrant patient participants to the study between May 2020–September 2021 from seven GP practices. Site 1 comprised 22 participants in six practices in Barnet, North London. Site 2 comprised 35 participants in one practice in Tower Hamlets, East London. Participants were followed up for up to 14 months in site 1 (median: 12 months, 1 participant followed up for 2 months only due to late recruitment) and for 6 months in site 2. We conducted 3 FGDs with a total of 30 practice staff (practice nurses, HCAs, and practice managers), in-depth interviews with two practice staff (lead practice nurse and assistant practice manager), and 39 in-depth interviews with migrants.

### Sociodemographic description of migrant participants

The mean age of the combined study population was 41 years (SD: 7.2 years); 62% were female. Participants had spent a mean 11.3 years (SD: 9.1 years) in the UK at the time of recruitment and came from 18 different countries of birth across Eastern Europe, Africa, Latin America and the Caribbean, and Asia, although the majority (75%) were born in Asia. Sixteen percent of participants had a vaccination card. Half (50%) of participants from site 1 migrated to the UK for economic reasons, with smaller proportions reporting forced migration, study, or joining/accompanying family. Two thirds (64%) of participants from site 1 were currently working in higher-skilled jobs (based on ONS Labour Force Survey categories [28]). Data on migration reasons and occupation were not collected from site 2. Demographic distributions did not differ statistically significantly between sites for age, sex, or years in UK but did differ by birth region (*p* = 0.01) and possession of vaccination card (*p* = 0.009) (Additional file 1: Table S1). Sociodemographic characteristics are shown in Additional file 1: Table S2.

### Under-vaccination for routine and selective/travel vaccines at study outset

**Fig. 3:**
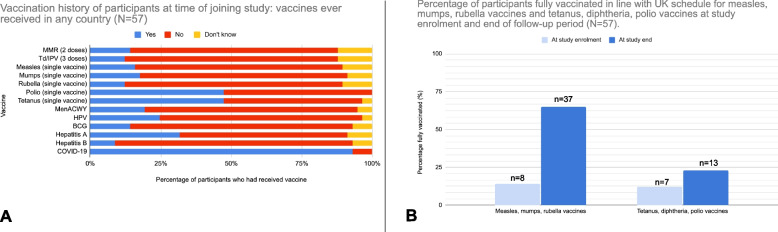
**A** Vaccination history at the time of joining the study (*n* = 57). Uptake of complete dosages shown for MMR (2 doses) and Td/IPV (3 doses); history of receiving at least one dose shown for all other vaccines. **B** Percentages of participants fully vaccinated in line with UK schedule for measles, mumps, and rubella vaccines and tetanus, diphtheria, and pertussis vaccines at study enrolment and end of follow-up period (*N* = 57)

A high proportion of our study population were incompletely vaccinated or unvaccinated according to UK immunisation guidelines [21] for several routine vaccines (Fig. 3; Additional file 1: Table S3). Specifically, 86% [29] of participants were incompletely vaccinated or unvaccinated for measles, mumps, and rubella vaccines and 88% [30] for diphtheria, tetanus, and pertussis vaccines (Table 2). Forty-seven (82%) participants had never received any measles-containing vaccine (single or combined vaccines), and 27 (47%) participants had never received any polio-containing vaccine.

**Table 2:** Participants assumed un-immunised and fully vaccinated in line with UK schedule for measles, mumps, rubella, tetanus, diphtheria, and polio at time of joining study and at study measles, mumps, rubella, tetanus, diphtheria, and polio at time of joining study and at study end (*N* = 57)

Diseases	At time of joining study		At study end	
	Assumed unimmunised, *n* (%)	Fully vaccinated in line with UK schedule, *n* (%)	Assumed unimmunised, *n* (%)	Fully vaccinated in line with UK schedule, *n* (%)
Measles, mumps, and rubella	49 (86%)	8 (14%)	20 (35%)	37 (65%)
Tetanus, diphtheria, and polio	50 (88%)	7 (12%)	44^a^ (77%)	13^a^ (23%)

Participants were asked about their history of receiving combined and single vaccines. *Assumed unimmunised* = uncertain, missing, or incomplete vaccination history. *Fully vaccinated at study end* includes those who were fully vaccinated at time of joining study plus those who became fully vaccinated by receiving catch-up vaccination

^a^Up to five more participants may have been fully vaccinated at study end (and therefore not ‘assumed unimmunised’); however, it is not possible to report it with confidence due to method of collecting data, where history of 1 or 2 previous doses of Td/IPV were coded as one variable

Among selective vaccinations (offered according to criteria such as age), 14 (25%) participants had received an HPV vaccination in any country, and 11 (19%) participants had received MenACWY vaccination in any country (Additional file 1: Table S3); neither of the 2 (4%) participants who were currently eligible for these vaccinations based on age had received either vaccine at the time of joining the study.

In addition, 86% reported never having received a TB/BCG vaccine, and 91% had never received a hepatitis B vaccine. Migrants reported high levels of COVID-19 vaccination: most (53, 93%) had received at least one dose of the COVID-19 vaccine (54% had received 3 doses, 35%—2 doses; 4%—1 dose) (Additional file 1: Table S4).

### Factors associated with under-vaccination for key vaccines

Factors associated with under-vaccination for key vaccines are not reported here (available via Additional file 1: Table S5) due to characteristics of the sample (very small number of participants who were fully vaccinated) rendering the findings inconclusive.

### Vaccinations offered and received in the UK (prior to study participation)

All participants reported having been offered vaccines in the UK, and 88% said that they had received something. The most offered vaccines were COVID-19 vaccine (100%), influenza vaccine (51%), hepatitis A (21%), and typhoid vaccine (18%). Less than 10% of participants were offered any of MMR, Td/IPV, MenACWY, and HPV, which are catch-up vaccines as per UK guidelines (Additional file 1: Table S2).

### History of VPDs in presenting migrants

History of VPDs was collected from site 1 participants (and both sites for COVID-19 disease) and is shown in Additional file 1: Table S6. Half of patients from site 1 (12 [55%] of 22 participants) recalled having a VPD (exact timing unknown and not including COVID-19), and 3 (14%) participants reported having had two or more VPDs (not including COVID-19). Reported VPDs included measles (*n* = 2), rubella (*n* = 1), active TB (*n* = 1), bacterial meningitis (*n* = 1), pertussis (*n* = 2), hepatitis A (*n* = 1), hepatitis B (*n* = 1), and HPV (*n* = 5) (Additional file 1: Table S6). Fifty (88%) of 57 participants reported having had COVID-19 disease (Additional file 1: Table S6).

### Uptake of catch-up vaccinations

The aggregated data show that 53 (93%) participants were referred for catch-up vaccinations as part of the study (Additional file 1: Table S7). Three quarters (43, 75%) received at least one dose of any catch-up vaccination. The most common reason for not receiving at least one dose was loss-to-follow-up from not responding to the invitation (5/10, 50%). Table 2 and Fig. 3B shows the proportion of participants who were fully vaccinated in line with the UK schedule for measles, mumps, and rubella vaccines and tetanus, diphtheria, and polio vaccines at the time of joining the study and at the end of the follow-up period.

Of the 52 participants referred for MMR (including one who was fully vaccinated at study start but received additional boosters through the study), 33 (64%) completed their required course (determined based on individual history and UKHSA algorithm for catch-up vaccination [21]). Although our study was not powered to statistically compare the differences between sites, 33% [6] of referred participants in site 1 had completed their required course of MMR by study end, compared to 84% [27] in site 2 (Table 2).

Fifty-one participants were referred for Td/IPV vaccination (including 3 who reported being fully vaccinated at study start but were referred for additional boosters). By the end of the follow-up period, 6 (12%) participants had completed their required course of Td/IPV, 40 (78%) had not, and 5 (10%) were unclear (due to limitations in the recording of their initial vaccination history data, but may have completed their course) (Table 2). There were again differences noted between sites (Additional file 1: Table S7).

Two (4%) participants were eligible to be referred for MenACWY vaccine and two (4%) for HPV vaccination based on age. Both eligible participants were referred for MenACWY vaccine and one participant (50%) received one dose. One (50%) of the two eligible participants was referred for a HPV vaccine but did not receive it (Additional file 1: Table S7).

### Qualitative findings

**Fig. 4:**
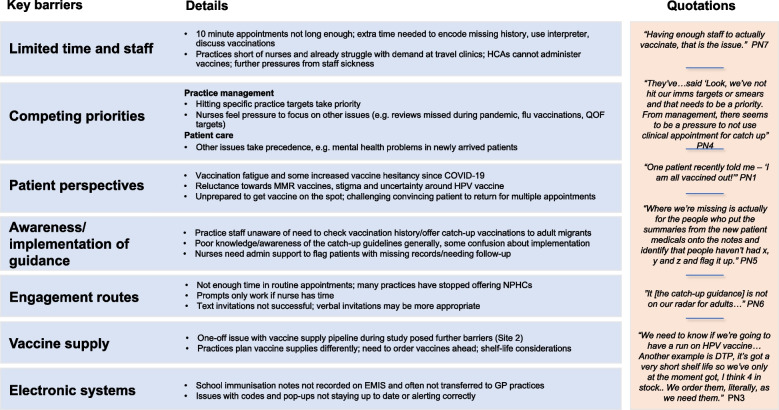
Key barriers to delivering catch-up vaccination in primary care with illustrative quotations, taken from 3 focus group discussions with 30 practice nurses/staff and 1 in-depth interview with a practice nurse and assistant practice manager

Through our FGDs with practice staff, we identified 7 key barriers to delivering catch-up vaccination in primary care (Fig. 4). Staff offered several suggestions to strengthen delivery of catch-up vaccination in primary care, which we grouped into 7 main areas for improvement and support (Table 3). Suggested levels of responsibility (practice, system, and policy) for changes are also highlighted. The in-depth interviews with migrants highlighted a range of barriers to catch-up vaccinations, including rarely being offered catch-up vaccination upon arrival or upon presenting to a healthcare facility, as well as factors related to trust, safety, and side effects, and preferences for natural immunity (full qualitative dataset reported elsewhere [26]).

**Table 3:** Improving catch-up vaccination: synthesis of staff suggestions and recommendations generated in FGDs

**Strengthen and clarify existing guidance and data**
• Clearer, more specific guidance needed; decision trees for specific scenarios may help; involve staff in co-designing updated catch-up vaccination algorithm **(policy)** • Unify European and UK catch-up guidelines to advance progress towards regional goals and ensure migrants are aligned with core schedule of vaccinations **(policy)** • Introduce clear mandatory and statutory governance of a multi-disciplinary integrated immunisation leadership structure **(policy)** • Routinely record migration status in electronic patient records and proactively check immunisation history of migrant patients attending primary care to identify those requiring catch-up vaccination. **(policy)**
**Explore and evaluate novel pathways, settings, approaches and funding mechanisms**
• Book new migrant patients in for immunisation reviews upon registering with a practice **(practice)** • Trial novel engagement routes for catch-up vaccinations and innovative financing mechanisms to support delivery and implementation, e.g. designated clinics, longer and out-of-hours appointments, nurse-led community interventions, school- and work-based awareness raising and signposting **(practice/system/policy)** • Identify and implement measures to reduce pressures in primary care and/or explore feasibility of shifting responsibility for catch-up vaccinations outside of primary care **(policy/system)**
**Set and use targets and incentives**
• Introduce government-backed catch-up vaccination targets and financial incentives in general practice; ensure immunisation targets and indicators are incorporated into integrated care strategies **(policy)** • Use existing pop-ups and available data to identify potentially eligible patients opportunistically **(practice)**
**Facilitate and champion good practice**
• Normalise checking vaccination history and offering catch-up vaccination to migrant patients **(practice/system)** • Identify staff champions to motivate, lead and inspire staff to meet targets **(practice/system)** • Promote and establish mechanisms of shared working across primary care networks through infrastructure funding, IT systems, governance, shared flexible staffing, and HR agreements** (practice/system)**
**Provide training**
• Train clinical staff in motivational interviewing and conversational techniques to encourage vaccination uptake; provide training resources, e.g. speaking to vaccine hesitant patients and examples of answers to challenging questions; deliver training on migrant health needs and cultural competency **(practice/system)** • Implement clear, appropriately funded immunisation training pathways for healthcare professionals and continue to identify and expand primary care groups able to carry out immunisation training (e.g. pharmacists), enabling less experienced healthcare professionals to focus on delivery of less complex immunisation programmes and freeing experienced staff to focus on more complex areas, e.g. catch-up vaccination **(policy/system)**
**Tailor services**
• Understand patient demographics **(practice)** • Use carefully considered wording and formats to invite patients and explain vaccination needs and opportunities **(practice)** • Employ staff of similar cultural/linguistic backgrounds as patient population or create designated roles in primary care focused on community engagement and reducing barriers to services for marginalised groups **(practice/system/policy)** • Utilise place-based partnerships (including NHS, local council, community and voluntary sector, local residents, service users, carers, representatives and community partners) to co-design and deliver integrated services to strengthen catch-up vaccination locally **(practice/system/policy)**
**Designate adequate funding and infrastructure**
• Expand funding and resources for routine childhood immunisations programmes to enable recall outside of the standard age groups for catch-up vaccination of adults and adolescents **(policy/system)** • Ensure adequate funding for primary care nursing and community nursing workforce sustainability **(policy/system)** • Reconcile issues with electronic/IT systems to ensure linkage between schools and GP immunisation records and wider system engagement **(practice/system)** • Consider the roles of integrated care systems (ICSs) in delivering recommendations and improving quality, efficiency, equity, and outcomes **(system)**

**Fig. 5:**
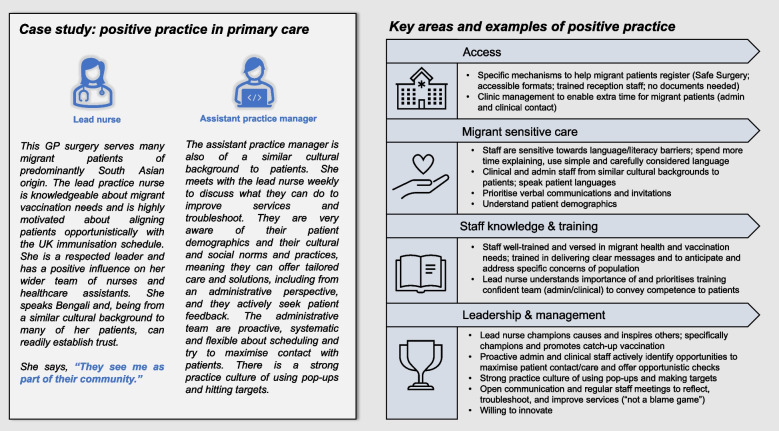
Case study of positive practice supporting catch-up vaccination in primary care, taken from focus group discussion with most successful participating GP surgery

A case study of positive practice supporting catch-up vaccination of adult migrants in primary care based on findings from the key informant interview conducted with staff from site 2 is highlighted in Fig. 5.

## Discussion

We engaged a diverse group of migrants to the UK, the majority (> 86%) of whom had incomplete or uncertain immunisation history for core vaccines in the UK immunisation programme, including MMR and Td/IPV, and had not previously been offered catch-up vaccination in the UK. This finding supports existing evidence suggesting that migrants are an under-vaccinated group in the UK that could benefit from catch-up vaccination on arrival [3, 6, 10].

### Implementation of the tool and referral pathways

When catch-up vaccination was facilitated by study teams, 53 (93%) participants identified as under-immunised were referred. However, although 43 (81%) participants had received at least one dose of a required vaccine at follow-up, only 6 (12%) referred for Td/IPV, and two thirds (33, 64%) of those referred for MMR had completed their required course and vaccination pathway at follow-up, suggesting that there were a range of individual and systemic obstacles to migrants accessing vaccinations (and multiple doses) that need to be better considered. Staff reported that there is rarely time in a routine appointment to engage migrants and offer catch-up vaccinations, and limited time to follow-up patients to invite them for catch-up, which may require multiple doses over an extended timeframe—especially when trust, language, and literacy barriers must also be overcome. Although the majority of patients attended a facilitated appointment with the practice nurse after being identified as requiring catch-up vaccination, drop out appears to have occurred for subsequent doses. There were also variations in interpretation of the guidelines, with some nurses recommending a full course of vaccinations regardless of previous history and others recommending only the doses or boosters needed to complete a previously started course. This ambiguity in the catch-up vaccination guidelines/algorithm was also mentioned in the FGDs and is likely to be approached differently between practices and practitioners, indicating a need for clearer, more standardised procedures to support implementation in future.

We found that site 2 was more successful than site 1 at starting participants on the catch-up vaccination pathway and facilitating further doses (100% started the pathway in site 2 compared to 44.4% in site 1, at end of follow-up period). This may be partly attributed to the positive influence of the lead nurse in site 2, who championed catch-up vaccination among her team, was aware of the vaccination needs of her migrant patients and the catch-up guidelines, and prioritised administering first doses immediately. This may also explain why 85% [27] of participants referred for MMR in site 2 had completed their required course at the end of follow-up, compared to 33% [6] in site 1. Some practices mentioned challenges with vaccine supply, and not being able to anticipate vaccine demand for adult patients, as this is not currently factored into orders. If government targets around adult catch-up vaccination were introduced, staff may become more likely to prioritise catch-up vaccination processes routinely, and practices may be better able to anticipate vaccine supplies. We saw comparable (but notably low) proportions of participants starting the Td/IPV pathway between sites, but lower completion of the pathway in site 2. This may be explained by a vaccine supply issue at site 2 at the time of the study, which was highlighted by the lead nurse during the key informant interview. This meant that the practice prioritised MMR vaccinations while they waited for replenished Td/IPV vaccine stock.

Our findings showing low uptake of subsequent doses of catch-up vaccines also suggested that efforts may be needed to address some vaccination concerns, hesitancy, and fatigue (post-COVID-19) and build trust with certain migrant groups [31]. This aligns with a recent systematic review which found acceptance barriers were mostly reported in Eastern European and Muslim migrant groups around HPV, measles, and influenza vaccines [12]. Another review highlighted sociodemographic and sociocultural barriers to HPV vaccination uptake [32]. Providing primary care staff with specific training in motivational interviewing techniques (an evidence-based approach to behaviour change) to facilitate positive conversations around vaccination and encourage uptake is effective in some settings and for some vaccines. It has been made available to NHS Wales staff [33–35] and may be an important component of any future implementation efforts. Building trust through an ‘insider’ perspective [36], for example through a personalised approach and involving a trusted health professional from a shared community, may be more effective than approaches involving a person perceived as an ‘outsider’ by the patient.

### Recommendations and considerations for strengthening the delivery of catch-up vaccination to migrants

Our quantitative and qualitative findings combined point to several changes needed at policy, system, and practice levels to strengthen the delivery of catch-up vaccinations to under-vaccinated migrant groups. Notably, there is a need to strengthen and clarify existing guidance and promote catch-up vaccination of migrants and older age groups in primary care. With coverage of previously eliminated diseases, such as measles, on the decline, the NHS has recently launched a catch-up campaign for missed MMR vaccines [37], and it will be important that immunisation leads and healthcare professionals are aware of the need to include adult migrants are included in these efforts. While catch-up vaccination guidelines for patients with no record or history of immunisation are straightforward, specific steps for those with partial immunity (e.g. conferred by single doses of vaccines or recollection of having a VPD such as measles as a child) are less clear and sometimes open to interpretation. Involving staff in co-designing an updated catch-up vaccination algorithm that addresses specific areas of confusion, adding decision aids and instructions for implementation, particularly to aid in identifying patients who may have missed routine vaccination, and introducing targets and training for catch-up vaccination in practices with high proportions of migrant patients are approaches that could be considered. Practices will likely prioritise hitting incentivised targets set in the current Quality and Outcomes Framework (QOF), which does not include catch-up vaccinations, particularly if they are overstretched or understaffed. It is unlikely that vaccination for migrants will be included in future versions of the QOF but financial and administrative support for vaccination in this group could be provided by Integrated Care Boards in England as part of local contractual arrangements for general practice.

We found that identifying a staff champion who understands the guidance and can lead and motivate practice staff to deliver catch-up vaccination and meet targets was effective. Having practice staff from similar cultural and linguistic backgrounds as patients may help instil patient trust and improve the delivery of culturally competent care [38, 39], while staff may be more invested in supporting causes and goals that benefit their community, including catch-up vaccination. Establishing peer or patient vaccine champion schemes or other designated trust-building roles in primary care, particularly in highly diverse, deprived, or underserved geographical areas, may also be an effective way to build trust and encourage vaccine uptake within specific communities [40–42] and could be explored with local government, NHS, and public health teams’ support. Outreach and engagement with local migrant communities to understand needs, address health concerns, and share information to change perceptions and promote healthy behaviours around vaccination and other health topics could be done in partnership with local community and voluntary sector organisations.

Even with stronger guidance and mechanisms to prompt staff to consider catch-up vaccination, barriers to delivery will remain due to intense pressures in primary care and wide variations in context which can affect implementation in practice [43]. Our data suggest that it may be more effective to explore and evaluate novel pathways, settings, and community-led or community-based outreach and interventions to deliver catch-up vaccination and innovative financing mechanisms to support delivery and implementation. These could include, for example, offering catch-up vaccination through the New Patient Health Check or NHS Check in primary care, where there is more time to discuss preventative health care, conducting immunisation reviews for new patients, and using community settings, peer- or nurse-led interventions in the community, reducing or removing the burden from primary care. During the COVID-19 pandemic and the more recent London-based polio booster campaign, multiple innovations were seen in the delivery of vaccinations to marginalised groups, including offering incentives and flexible arrangements and infrastructure (e.g. out-of-hours clinics and alternative settings for vaccination [44–48]. How these approaches could be used for delivering routine immunisations to migrants must be considered [49], ensuring the involvement and support of these communities in research and policy decisions. Working in partnership with community assets and networks to provide more localised and flexible approaches and outreach has been effective at facilitating attendance at NHS Health Checks [29] and should be explored. Ensuring migrants are involved in co-designing interventions that address their needs will also be vital [30].

### Novel contributions to the literature

Our findings align with much of the wider literature documenting the under-immunisation of migrants in Europe [1, 3, 5, 50, 51]. We have uncovered similar barriers to delivering adult migrant catch-up vaccinations as reported in studies done in the UK, Australia, and Norway [10, 52, 53], including a lack of consistent guidelines, gaps in training and knowledge leading to missed opportunities by service providers, and perceptions that catch-up vaccination is time-consuming, difficult, and resource intensive. Our study builds on this work through its practical efforts to pilot test a standardised data collection tool and referral pathway to facilitate vaccine delivery in primary care, and through its mixed-methods approach, which allowed for triangulation of data, a case study of good practice, and formulation of evidence-based recommendations. However, efforts are also needed to unify regional policy and increase the inclusion of adult migrants at the regional level. Evidence shows that policies and practices differ in European countries with respect to adult vaccination and the inclusion of adult migrants in vaccination programmes on arrival [4, 54, 55]. For example, only 13/32 countries in the EU/EEA had policies in place to offer MMR to adult migrants (10 countries said they would charge fees). Addressing barriers at a regional level will be particularly important for meeting ECDC and WHO objectives to increase vaccine access and equity and ensure the integration of refugees and migrants in immunisation policies, service delivery and planning globally [15, 19, 56].

### Strengths and limitations

A strength of this study was its novel approach to assess under-vaccination and align migrant patients with the UK immunisation schedule, reducing their risk of contracting VPDs or experiencing ill health and closing immunisation gaps. Conducting this study during the COVID-19 pandemic, however, was challenging, with resource constraints and competing priorities in primary care posing additional complexities to implementation, as well as the need for modifications to our recruitment and referral pathways in some participating GP practices, a much longer study period than we anticipated with low recruitment, and a shortened follow-up period in site 2. However, these challenges reflect realities of primary care and led to valuable findings from our pilot study that can be used to inform future strategies and implementation. We also had major challenges recruiting patients in some practices, partly due to the poor recording of migrant status in electronic medical records, making it difficult to identify our target population, and due to very high work loads of practice nurses and CRN staff for the duration of our study. Low recruitment may also be reflective of migrant patients’ ‘vaccine fatigue’, reluctance to attend health facilities during the COVID-19 pandemic, or heightened mistrust or anticipated stigma [57–59] which increased their reluctance to receive or discuss vaccinations or engage with our study. The small sample size makes it difficult to generalise findings or draw wider conclusions, but this pilot will inform future large-scale studies. The extent of under-vaccination among this random sample, coupled with the qualitative data emphasising the absence of effective pathways for offering catch-up vaccination to these groups through primary care, serve as preliminary evidence of a public health concern that merits further research and understanding.

## Conclusions

In conclusion, our pilot study indicates that adolescent and adult migrants in the UK may be under-vaccinated and require catch-up vaccination. Addressing this gap requires implementing effective pathways in primary care, which could be supported by designated staff champions, training, awareness campaigns, and financial incentives. Community-based approaches to delivering catch-up vaccination among these populations should also be explored.

### Supplementary Information

**Additional file 1: Table S1.** Comparisons of distributions of sociodemographic values between study sites (site 1, *N* = 22; site 2, *N* = 35), performed using Pearson’s Chi-squared test and unpaired t-tests. **Table S2.** Sociodemographic characteristics of study population, by site and combined. **Table S3.** Study participants’ vaccination history at time of recruitment. **Table S4.** Participants’ COVID-19 vaccination offers and uptake in the UK. **Table S5.** Logistic regression analyses showing factors associated with un-vaccination for polio (zero doses of single or combined vaccines). **Table S6.** Participants’ history of VPDs at time of study recruitment. **Table S7.** Catch-up vaccination referral and uptake as part of the study.

**Additional file 2.**

## Data Availability

Data are available on reasonable request from researchdata@sgul.ac.uk.

## Abbreviations

BCG: Bacille Calmette-Guerin
COVID-19: Coronavirus disease
CRN: Clinical research network
ECDC: European Centre for Disease Prevention and Control
FGD: Focus group discussion
GP: General practice/general practitioner
HCA: Healthcare assistant
HPV: Human papillomavirus
IA2030: Immunization Agenda 2030
IOM: International Organization for Migration
MenACWY: Meningococcal ACWY vaccine
MMR: Measles, mumps, rubella vaccine
NHS: National Health Service
NIHR: National Institute for Health and Care Research
NoCLoR: NHS North Central London Research Network
ONS: Office for National Statistics
PICOTS: Patient, intervention, control, outcome, time, study design
PPIE: Patient and Public Involvement and Engagement
QOF: Quality Outcomes Framework
SD: Standard deviation
STROBE: Strengthening the Reporting of Observational Studies in Epidemiology
TB: Tuberculosis
Td/IPV: Tetanus, diphtheria, polio vaccine
UKHSA: UK Health Security Agency
VPD: Vaccine-preventable disease
WHO: World Health Organization
