# Supplementary material for: Driving delivery and uptake of catch-up vaccination among adolescent and adult migrants in UK general practice: a mixed methods pilot study
Source: BMC Med. 2024 May 3;22:186. doi: 10.1186/s12916-024-03378-z (PMC11068568; doi:10.1186/s12916-024-03378-z)
Supplement: Supplementary file 2 — Additional file 2. [file 12916_2024_3378_MOESM2_ESM.pdf]

	Albanian	Bengali	Hindi	Polish	Romanian	Somali	Turkish	Urdu
**MMR**	FRP	এমএমআর<br>MMR3/MMRV	(एमएमआर -MMR)<br>वैक्सीन - MR<br>mumps vaccine not given	MMR	ROR		KKK	n/a (given 2 measles vaccines (9 & 15M)
**Measles**	Fruthi	হাম (মিজেলস)	खसरा / मीजल्स	Odra,	Rujeolei/ Pojar	Jaceecada	Kızamık	خسره
**Mumps**	Shyta	**মাম্প্স** বা **পনসিকা**	मम्प्स	Swinka	Oreionului	Qaamow-<br>Qashiirta	Kabakulak	ممپس
**Rubella**	Rubeolë	রুবেলা	रूबेला	Różyczka	Rubeolei,	Jadeeco<br>jarmalka/<br>Rubellaha	Kımamıkçık	روبیلا
**DTP**	DTP	ডপট (DPT)	डीपीटी	DTP	Di-Te-Per/		DaBT	
**Diptheria**	Difteri	ডিপথেরিয়া	**रोहिणी** या<br>**डिप्थीरिया**	Przeciwko blonicy,<br>Blonica	Difteriei	Gowracato	Difteri	ڈپتھیریا (خناق)
**Tetanus**	Tetanoz	টিটেনাস	**टिटनेस** या **धनुस्तंभ**	Tezec, Tężcowi	Tetanosului,	Teetanada	Tetanos	ٹیٹنس<br>تشنج
**Pertussis**	Pertusis	হুপিং কাশি (পারটুসিস)	काली खांसी<br>(कुक्कुर खाँसी)	Krztusiec/<br>krztuścowi	Tuse convulsiva	Xiiqdheer	Boğmaca	کالی کھانسی<br>(پرٹوسس)
**Polio**	Poliomielit	পোলিও	पोलियो	Poliomyelitis (IPV)	Poliomielitei<br>(VPO)	Dabayl/<br>dabaysha	Çocuk Felci	پولیو<br>(آئی پی وی)
**BCG/ TB**	Tuberkuloz<br>(BCG)	বিসিজি	बी॰सी॰जी॰ (**यक्ष्मा**)<br>(BCG)	Gruźlicy	Tuberculozei<br>(BCG)	Tallaakla<br>Qaaxada	BCG (verem/<br>tüberküloz aşısı)	بی سی جی<br>(تپ دق)<br>(BCG)
**Meningitis ACWY**	Meningokokut<br>ACWY	ম্যানানজাইটিস ACW	**तानिकाशोथ** या<br>**मस्तिष्कावरणशोथ**<br>ACWY	Meningokokum<br>ACWY	meningococic<br>ACWY	Qoorgooyaha<br>ACWY	Meningokokal<br>ACWY	ACWY میننجائٹس
**Hepatitis B**	Hepatit viral B	হেপাটাইটিস B	हेपेटाइटिस बी	Wirusowe<br>zapaleniu<br>wątroby typu B	Hepatitei virale<br>B	Cagaarshow<br>nooca B	Hepatit B	ہیپاٹائٹس بی
**Hepatitis C**	Hepatit viral C	হেপাটাইটিস C	यकृतशोथ ग<br>(हेपेटाइटिस सी)	Wirusowe<br>zapaleniu<br>wątroby typu C	Hepatitei virale<br>C	Cagaarshow<br>nooca C	Hepatit C	ہیپاٹائٹس سی
